# Why *Hereditas* leads hereditary cancer awareness now more than ever

**DOI:** 10.1186/s41065-025-00556-8

**Published:** 2025-09-29

**Authors:** Julhash U. Kazi, Ramin Massoumi

**Affiliations:** Division of Translational Cancer Research, Department of Laboratory Medicine, Lund University, Lund, 22363 Sweden

*This Editorial was written in recognition of Hereditary Cancer Awareness Week (Sunday*,* September 28 to Saturday*,* October 4*,* 2025) to highlight Hereditas’ contributions to research and progress in hereditary cancer. These efforts support discovery*,* innovation*,* and the advancement of Sustainable Development Goal 3: Good Health and Wellbeing. Commissioned by the Publisher*,* India Sapsed-Foster*,* the Editorial was co-authored by Hereditas’ Editor-in-Chief*,* Dr. Ramin Massoumi*,* and Editorial Board Member*,* Dr. Julhash U. Kazi—both from Lund University.*

**Figure:**
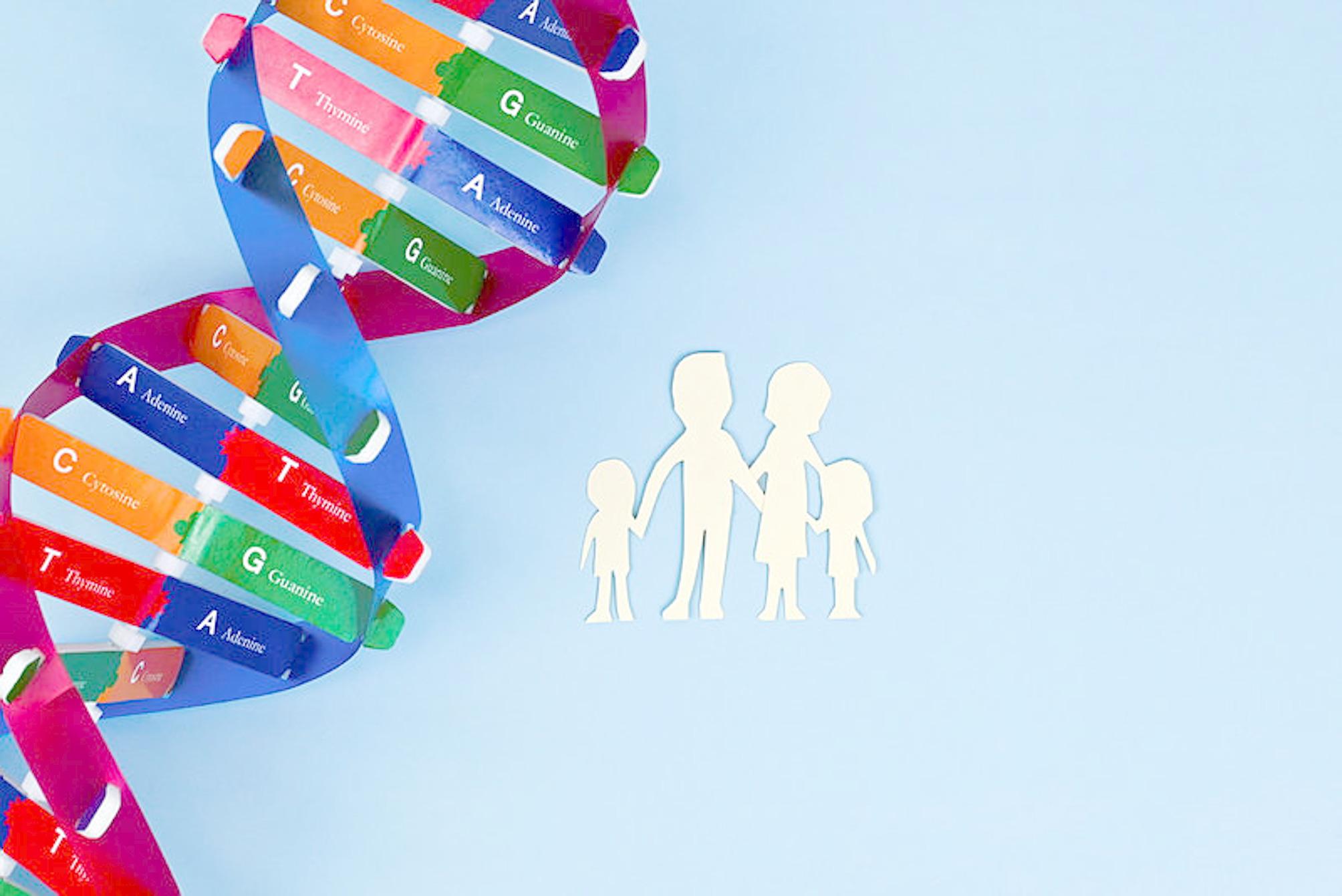
Image: ©joy/Stock.adobe.com

## Summary

Hereditary cancers remind us that genetics is never just about science; it is about people, families, and futures. What begins as a discovery in the laboratory can change how a parent makes decisions about screening, how a clinician guides treatment, or how a community thinks about prevention. Today, advances in gene therapy, RNA-based medicine, biomarkers, and precision tools are expanding what is possible, but their true value lies in how they improve lives. At *Hereditas*, we see our role as more than curating research. It is about connecting breakthroughs to the wider world. In this commentary, we highlight examples of work published in the journal that demonstrate how discoveries in genetics and genomics can translate into clinical practice and societal benefits. Awareness weeks provide an important reminder. The advances we publish today are not only milestones in genetics, but also the seeds of tomorrow’s prevention and cures.

## Hereditas and the responsibility of awareness

Hereditary cancers are where science meets lived experience. For more than thirty years, since the discovery of BRCA1/2, genetics has given us new ways to identify risk, intervene early, and ultimately save lives. But progress in the laboratory is not enough. It must be connected to the people who need it most, families living with hereditary risk, clinicians guiding prevention, and communities who may not yet be aware of the role their genes play in health. At *Hereditas*, with our century-long tradition of publishing advances in genetics, we have always seen awareness as part of our responsibility. And today, in the era of genomic medicine, that responsibility is greater than ever. As stated in an earlier editorial, the pace of change in genetics is unlike anything seen before. Tools such as CRISPR, next-generation sequencing, and precision medicine “promise to revolutionise healthcare by offering solutions to some of the most pressing challenges facing humanity” [1]. The question is not whether these breakthroughs will change medicine, but how quickly these insights reach the people and communities who need them most. Awareness is the bridge.

## Genetics at the heart of modern oncology

Genetics is cancer care, since DNA testing is central to how we prevent, diagnose, and treat malignancies [2]. Families with hereditary mutations, such as BRCA1/2, now routinely benefit from early screening and preventive surgery. Oncologists rely on genetic profiling to guide therapy choices, and researchers show that we may soon be able to correct inherited cancer-causing mutations at their source [3]. These changes are reshaping conversations in clinics, decisions in families, and the way health systems consider prevention. Artificial intelligence and multi-omics are accelerating this transformation, allowing treatment strategies to be adjusted dynamically to match the biology of each patient [4]. This is where journals like *Hereditas* have a vital role. We simply do not publish data; instead, we provide the data context, visibility, and a platform for dialogue. Our responsibility is to show how genetics moves from bench to community, from theory to impact.

## Examples from Hereditas: turning discovery into possibility

Our recent publications illustrate what this looks like in practice. We have seen cancer therapy shift from blunt chemotherapy toward personalized strategies, while new studies describe how targeted drugs, immunotherapies, mRNA vaccines, and AI-guided omics are emerging to overcome resistance and tailor treatment to the individual [5, 6]. These are not isolated findings instead, they reflect that genetics is not only deepening our understanding of cancer but also expanding the available tools to those on the frontlines of care. Take the telomerase gene DKC1, which is long known from the hereditary disorder dyskeratosis congenita. A 2023 study in *Hereditas* revealed that DKC1 is frequently overexpressed across cancers, and laboratory work showed that silencing this gene halted proliferation and migration in tumor cells [7]. What began as a rare hereditary syndrome now informs our understanding of cancer biology more broadly. Another example is the circular RNA circHMCU, which was investigated in breast cancer. The study demonstrated that circHMCU drives tumor growth through functional suppression of miR-4458, a tumor-suppressive microRNA. Silencing circHMCU restored miR-4458 activity and inhibited cancer progression both in vitro and in vivo [8]. In another study, Zheng and colleagues reported on an engineered measles virus (rMeV-Hu191) with selective oncolytic activity against breast cancer. In preclinical models, this virus induced tumor apoptosis and suppressed growth without harming healthy tissue [9]. The idea that a virus, reprogrammed by modern genetics, could become a targeted therapy is a glimpse of what may soon be possible.

## Beyond the lab: broadening access and anticipating risks

Our responsibility does not end with the new therapies. Awareness also includes thinking about access, safety, and resistance. In gastric cancer, often diagnosed late, researchers have identified circulating lncRNAs such as PANDAR as promising biomarkers. This could support low-cost screening in community hospitals, where advanced imaging is unavailable [10]. If implemented, such assays could help reduce survival disparities tied to geography and resources. Prostate cancer offers another example. Work in *Hereditas* identified the lncRNA PSMA3-AS1 as a prognostic marker, elevated in tumors and linked to poor survival. Silencing it restored tumor-suppressive activity of miR-29a-3p and reduced malignant properties in cell models [11]. We also need to remain mindful of safety. Doxorubicin is a cornerstone of chemotherapy, yet its cardiotoxicity casts a long shadow. Recent findings discovered that USP8 overexpression in cardiomyocytes stabilizes MDM4 and reduces ferroptosis during doxorubicin exposure, pointing to potential cardioprotective strategies [12]. Such work reminds us that precision does not end when chemotherapy stops; survivorship is part of the continuum. Resistance to the therapy, often translated as a failure, is indeed a biological state to target. In gastric cancer, cisplatin sensitivity was enhanced by carnosic acid through a TP53-mediated pathway [13]. In hepatocellular carcinoma, EPHX1 was identified as a driver of regorafenib resistance [14]. In breast cancer, combining radiotherapy with PD-1 inhibitors not only improved progression-free survival but also reduced EGFR expression, with baseline EGFR serving as a prognostic factor [15]. Each of these studies reframes resistance as a challenge to be met with new strategies rather than a dead end.

## Why awareness matters

When we look across these contributions, a clear pattern emerges. Whether by identifying hereditary risk genes, advancing RNA-based therapies, or developing viral and immune-based treatments, *Hereditas* publications are united by a shared aim, which is translating genetics into practice. They are not only academically rigorous, but they are societally meaningful. That is why *Hereditas* leads hereditary cancer awareness now more than ever. It is also aligned with the United Nations Sustainable Development Goal 3 (SDG 3), ensuring healthy lives and promoting well-being for all at all ages. This represents a convergence of scientific progress and social justice [16]. Target 3.4 of SDG 3 specifically aims to reduce premature mortality from noncommunicable diseases, including cancer, by one-third by 2030. Early detection of cancer using suitable biomarkers, as well as monitoring disease progression and therapy resistance ; , are examples from *Hereditas* that may contribute to reducing mortality over time [10, 14, 15]. Awareness weeks are not simply markers on a calendar. They are opportunities to connect science with people and to remind us that the work in our journals and laboratories has a direct line to families making decisions about their health. However, with opportunity comes responsibility, which is to safeguard privacy, ensure equity, and engage dialogue about the promises and limits of genomics. At *Hereditas*, we see hereditary cancer awareness as an essential part of our mission. The advances we publish in gene therapy, genomic risk assessment, and targeted treatment are not only about the future of medicine; it is indeed about decision-making. By aligning our publications with this cause, we show how a scientific journal can influence change far beyond academia. The strategies and treatments we document today are the preventive measures and cures of tomorrow. This is why we lead hereditary cancer awareness now more than ever.
